# Evaluating the burden and transmission dynamics of chikungunya virus infections in the Eastern Mediterranean Region: a systematic review and meta-analysis

**DOI:** 10.1093/eurpub/ckae165

**Published:** 2025-01-13

**Authors:** Riyaz Ahamed Shaik, Mohammad Shakil Ahmad, Mohammad Miraj, Waqas Sami, Alashjaee Ahmed Azam, Patrick Okwarah

**Affiliations:** Department of Family and Community Medicine, College of Medicine, Majmaah University, Majmaah, Saudi Arabia; Department of Family and Community Medicine, College of Medicine, Majmaah University, Majmaah, Saudi Arabia; Department of Physical Therapy and Health Rehabilitation, College of Applied Medical Sciences, Majmaah University, Majmaah, Saudi Arabia; Department of Pre-Clinical Affairs, College of Nursing, QU-Health, Qatar University, Doha, Qatar; Department of Internal Medicine, College of Medicine, Jouf University, Saudi Arabia; Department of Community Health, Amref International University, Nairobi, Kenya; Infectious Hazard Prevention and Preparedness Unit, Department of Health Emergency, World Health Organization, Eastern Mediterranean Region Office, Cairo, Egypt

## Abstract

The Chikungunya virus (CHIKV) presents substantial public health challenges in the Eastern Mediterranean Region (EMR), with its prevalence and interaction with other arboviruses (ABVs) remaining poorly understood. This systematic review and meta-analysis aimed to assess the prevalence of CHIKV and its association with other ABVs, such as dengue virus (DENV), Rift Valley fever virus (RVFV), malaria, and yellow fever virus (YFV), in the EMR. We systematically searched databases including PubMed, Embase, Web of Science, Scopus, Cochrane Library, CINAHL, PsycINFO, and ScienceDirect to identify epidemiological studies that report CHIKV prevalence and provide odds ratios (ORs) for CHIKV compared to other ABVs. Data analysis was performed using a random-effects model. Heterogeneity was evaluated using the χ^2^ test and *I*^2^ statistic. The GRADE approach was used to evaluate the quality of the studies while the AXIS tool, NOS tool, and AHRQ checklist assessed the risk of bias. The meta-analysis revealed a significant prevalence of CHIKV in the EMR. However, the studies exhibited heterogeneity, indicating variability in the results. A comparison of CHIKV with other ABVs did not show any statistically significant differences in prevalence. The meta-analysis found a notable prevalence of CHIKV in the EMR. The results also indicated that the prevalence of CHIKV is comparable to that of other ABVs in the region. These findings provide an overview of the burden of CHIKV in the EMR.

## Introduction

Arboviruses (ABVs) are responsible for significant morbidity and mortality worldwide, each year [1–4]. Dengue virus (DENV) is among the most prevalent ABVs, causing a spectrum of illnesses from mild flu-like symptoms to severe dengue haemorrhagic fever, impacting millions globally [5]. Rift Valley fever virus (RVFV), which primarily affects domestic animals, also poses risks to humans, leading to conditions ranging from mild illness to severe complications like encephalitis and haemorrhagic fever [6]. Mosquitoes carrying RVFV have been linked to recurrent epidemics in Africa and the Arabian Peninsula [7]. Malaria is a major worldwide health concern because, if left untreated, it can result in severe sickness and even death [8, 9]. Yellow fever virus (YFV), carried by Aedes mosquitoes, can present with symptoms ranging from moderate febrile sickness to severe haemorrhagic fever and organ failure [10]. It is possible to receive a vaccination against YFV, which is essential for stopping outbreaks and halting the virus’s spread [11]. Assessing the costs associated with ABVs and developing efficient policies and resource allocation strategies are made more difficult by the complex social, economic, and healthcare system connections that are intertwined with the complicated epidemiology of these diseases [4].

### Chikungunya virus (CHIKV) in the Eastern Mediterranean Region (EMR)

Globally, CHIKV represents a serious public health issue, especially in areas where mosquito vectors are abundant [12]. Humans contract CHIKV mostly from the bite of an infected Aedes mosquito, which includes Aedes aegypti and Aedes albopictus. The virus is known for causing fever, rash, joint pain, and muscular aches, as well as inducing a febrile illness that can result in long-term consequences like arthritis and chronic joint pain [13]. The effects of CHIKV on economies, public health systems, and people’s quality of life highlight the necessity for in-depth study on the virus’s prevalence and routes of transmission.

The EMR, comprising 22 countries, is recognized for its susceptibility to the spread of ABVs due to its unique climate and biological characteristics [13]. This region engages in extensive international trade with neighbouring countries and has experienced rapid and unplanned urbanization, coupled with the proximity of human settlements to rural livestock-rearing areas, factors which collectively augment this vulnerability [14]. Because of its climate and biological characteristics, the EMR is recognized to be susceptible to the spread of ABVs [13]. The EMR has experienced repeated outbreaks of emerging infectious illnesses, which frequently result from differences in monitoring capacities and collaborative efforts at the intersection of human and animal health [1, 15]. Furthermore, nearly one-third of the world’s population lives in this region and is in need of aid; this situation is made worse by underdeveloped health systems and inadequate capacity for disease surveillance, readiness, and response [14–17]. Plans and interventions for public health must take into account the burden and dynamics of CHIKV transmission in the EMR.

### Study rationale and objectives

Previous studies, including a foundational review [2], have provided insights into the prevalence and interactions of CHIKV with other ABVs in the EMR. However, given the evolving nature of epidemiological factors such as climate change, urbanization, and population mobility, there is a critical need to update our understanding of these interactions and their implications for public health. This study aims to reassess CHIKV prevalence and its association with other ABVs in the EMR to inform the development of targeted interventions like vector control, early detection, and effective healthcare responses. By conducting this systematic review and meta-analysis, we seek to strengthen the regional strategies for CHIKV mitigation and support evidence-based policymaking for the control of arboviral diseases.

## Methods

### Review methodology

We conducted a systematic review using a structured methodology in accordance with the Preferred Reporting Items for Systematic Reviews and Meta-Analyses (PRISMA) guidelines [18], as depicted in Supplementary Fig. S1. This approach aimed to synthesize evidence on CHIKV infections in the EMR, thereby informing public health strategies and initiatives for effective disease prevention and control.

### PICOS protocol

The population of interest comprised individuals with CHIKV infections in the EMR region focusing on all age groups and both genders. Exposure to CHIKV was assessed through epidemiological, case–cohort, and prospective/retrospective studies examining its prevalence, incidence, or transmission in human populations. Comparisons were made between CHIKV and other ABVs where data was available. Studies comparing CHIKV prevalence across different countries within the EMR were also included. The comparative protocol was not mandatory, given the epidemiological nature of this investigation. The primary outcomes of interest were CHIKV infection prevalence, transmission dynamics, associated comorbidities, and its co-circulation with other ABVs. Eligible study designs included epidemiological (cohort, case–control, cross-sectional), case–cohort, and both prospective and retrospective studies that provided insights into CHIKV frequency, distribution, and risk factors in humans.

### Database search technique

A comprehensive search was conducted across PubMed, Embase, Web of Science, Scopus, Cochrane Library, CINAHL, PsycINFO, and ScienceDirect using a combination of MeSH terms, Emtree terms, and free-text keywords along with Boolean operators facilitating the construction of precise search algorithms tailored to each database’s unique indexing system. Supplementary Table S1 details the search strings that were utilized across each database. We focused on articles published between January 2000 and December 2023.

### Selection criterion

Studies were selected based on a predefined inclusion and exclusion criteria, the Patient, Intervention, Control and Outcomes (PICOS) framework (Supplementary Table S2) among other criteria. We included studies that documented human cases of CHIKV or genetic analyses of the virus and excluded those without specific epidemiological data or that focused solely on geographical distribution without human case data.

### Variable extraction strategy

Three reviewers independently screened each retrieved record and report. Data extraction strategy prioritized epidemiological research, case–cohort studies, and prospective/retrospective studies over those that only provided geographic data or lacked detailed information on the population under investigation. This approach was based on the need to gather comprehensive and reliable data about CHIKV infections in human populations. The selection was justified by the potential of these study types to offer accurate data on the incidence of CHIKV infections and associated risk factors. Epidemiological studies were included because they could provide a more profound comprehension of CHIKV prevalence and its impacts on human populations. Additionally, the use of case–cohort studies and the collection of prospective/retrospective data, as well as longitudinal follow-up, facilitated a better understanding of CHIKV infection dynamics over time. Geographic data alone were excluded unless it contributed to the understanding of transmission dynamics.

### Bias evaluation protocol

We applied specific bias evaluation techniques to ensure a consistent quality assessment across studies, as demonstrated in Supplementary Figs. S2 and S3. Prevalence studies were assessed using the Agency for Healthcare Research and Quality (AHRQ) checklist, while the Newcastle-Ottawa Scale (NOS) tool and the Appraisal tool for Cross-Sectional Studies (AXIS) were used to evaluate cohort-based and cross-sectional studies, respectively. These tools systematically measured potential biases within individual studies and were integral in maintaining a high standard of evidence synthesis and reducing the impact of possible biases on findings. The process of bias evaluation is illustrated in Supplementary Figs. S2 and S3.

### Certainty bias

The certainty of evidence and risk of bias in the included studies were evaluated using the Grading of Recommendations Assessment, Development, and Evaluation (GRADE) approach, in conjunction with the tools discussed in the previous section. The GRADE system was employed to evaluate the quality of the evidence from the included studies, which involved an examination of study limitations, consistency of results, directness of evidence, precision, and publication bias.

### Statistical analysis protocol

Meta-analyses were conducted using RevMan 5 (version 5.4.1) software. The random-effects (RE) model was employed to estimate the pooled prevalence of CHIKV across EMR countries (studies) and to compare the prevalence of CHIKV serotypes with other ABVs, including DENV, RVFV, malaria, and YFV. Odds ratios (ORs) were calculated for each comparison, accompanied by 95% confidence intervals (CIs) to assess the effect sizes. The RE model was used to account for variations in the study designs, techniques, and demographics between the different EMR countries. This allows for an estimation of the pooled prevalence of CHIKV while accounting for the between-study variability. The RE model is more conservative with estimates of the pooled prevalence and also takes into account the differences in the underlying population and the study characteristics as compared to the fixed-effects model. Forest plots were generated to visually compare the prevalence rates across different studies.

### Relevant ethical issues

Since our study synthesized existing published data and did not involve direct data collection from human subjects, specific ethical clearance from an institutional review board was not necessary. We ensured that the included studies had adhered to ethical standards and had obtained necessary approvals at the time of their original execution. We adopted a transparent methodology while minimizing bias, and ensuring responsible reporting of findings to uphold the ethical integrity of our review.

## Results

### Selection protocol

A systematic search strategy across multiple databases identified 738 potentially relevant papers. Following a structured screening process based on title and abstract reviews, and subsequent full-text assessments, 10 papers [19–28] were deemed eligible for inclusion in this review.

### GRADE assessment results

The quality of evidence from the nine cross-sectional studies was assessed using the GRADE approach summarized in Supplementary Table S3. Despite a low risk of bias, these studies exhibited low to moderate levels of inconsistencies and imprecision in outcomes related to CHIKV infection, with an overall moderate certainty of evidence. However, indirectness was low, and no additional factors affecting certainty were reported. The single prospective cohort study demonstrated a distinct outcome suggesting rapid recovery from CHIKV among subjects. The study presented a low risk of bias without any detected inconsistency, indirectness, or imprecision, leading to a moderate certainty of evidence. Detailed bias evaluations assessed across the studies are depicted in Supplementary Figs. S2 and S3.

### Variable description

As shown in Table 1, the included studies spanned several countries in the EMR: East Sudan [19, 26], Djibouti [20], Pakistan [21,22, 28], Saudi Arabia [24], Qatar [25], and Iran [27]. Sample sizes ranged from 40 to 5799 participants, with studies conducted over periods ranging from 4 to 36 months and subjects’ average age ranging from 27 to 36.6 years. Gender distribution varied, with some studies showing a balanced representation [23, 26], while others showed a predominance of one gender. The Djibouti study included 1045 participants divided into five age groups, with women overrepresented [20]. Three studies from Pakistan had sample sizes ranging from 584 to 1549, with an average age of about 31.8 years [21,22, 28]. The Saudi Arabian study did not provide information on age ranges, sample size, or gender distribution [24]. The Qatari study used a large sample size of 5799 participants, with only men employed [25]. The Iranian study had 159 participants, mostly men, with no age information provided [27].

**Table 1.** CHIKV-related variables observed in the included articles

Study ID	Year	Region	Sample size (*n*)	Age groups	Study design	Assessment period (months)	Demographics	CHKV assessment methods	Total CHKV prevalence	Associated comorbidities
Ali et al. [19]	2022	East Sudan	93	Unspecified	Cross-sectional	4	31.6 ± 3.4, all females	ELISA (IgM/IgG) and RT-qPCR	0.7%	None
Andayi et al. [20]	2014	Djibouti	1045	≤19; 20–39; 40–59; ≥60	Cross-sectional	4	Unspecified, 474 males	ELISA (IgG) and RT-qPCR	2.8%	DNV, malaria, RVV, YFV
Badar et al. [21]	2021	Pakistan	1549	0–10; 11–20; 21–30; 31–40; 41–50; ≥50	Cross-sectional	31	31.8 ± 15.7, unspecified	ELISA and RT-qPCR	50%	None
Badar N et al. [22]	2020	Pakistan	584	≤10; 11–20; 21–30; 31–40; 41–50; ≥50	Cross-sectional	6	31.8 ± 15.7, 277 males	ELISA (IgM) and RT-qPCR	70.3%	None
Bower et al. [23]	2021	East Sudan	142	≤2; 2–4; 5–14; 15–29; 30–49; ≥50	Prospective cohort	4 (follow-up)	27 ± 17.6, 74 males	ELISA (IgM/IgG) and RT-qPCR	84.5%	Malaria, DNV
Hakami et al. [24]	2021	Saudi Arabia	40	Unspecified	Cross-sectional	Unspecified	Unspecified, unspecified	ELISA (IgG) and RT-qPCR	2.5%	None
Humphrey et al. [25]	2019	Qatar	5799	≤24; 25–29; 30–34; 35–39; 40–44; 45–49; ≥50	Cross-sectional	36	36, all males	ELISA (IgG) and RT-qPCR	4.11%	DNV
Nahla et al. [26]	2019	East Sudan	119	Unspecified	Cross-sectional	Unspecified	31.98, 68 males	ELISA (IgM) and RT-qPCR	73.1%	HEV, malaria, RVV, YFV
Pouriayevali et al. [27]	2019	Iran	159	Unspecified	Cross-sectional	14	36.6 ± 15.8, 81 males	ELISA (IgM/IgG) and RT-qPCR	25.1%	Arthritis
Raza et al. [28]	2021	Pakistan	291	≤15; 16–30; 31–45; 46–60; ≥60	Cross-sectional	24	30 (median), 222 males	ELISA (IgM/IgG) and RT-qPCR	11.8%	DNV, NCDs, HTN, T2DM

### CHIKV-based assessments

We analysed the data of each study to understand the patterns and implications of CHIKV in the EMR. The studies utilized enzyme-linked immunosorbent assay (ELISA) and reverse transcription quantitative polymerase chain reaction (RT-qPCR) methods to assess CHIKV presence. ELISA was used to identify the CHIKV antibodies (IgM/IgG) and RT-qPCR was used to identify the CHIKV genes. The reported prevalence of CHIKV varied from 0.7% to a high of 84.5%. This suggests that prevalence estimations could be impacted by several variables, such as the study population, surrounding circumstances, and the degree of sensitivity of the assessment procedures employed.

### Regional analysis

**Figure 1.**
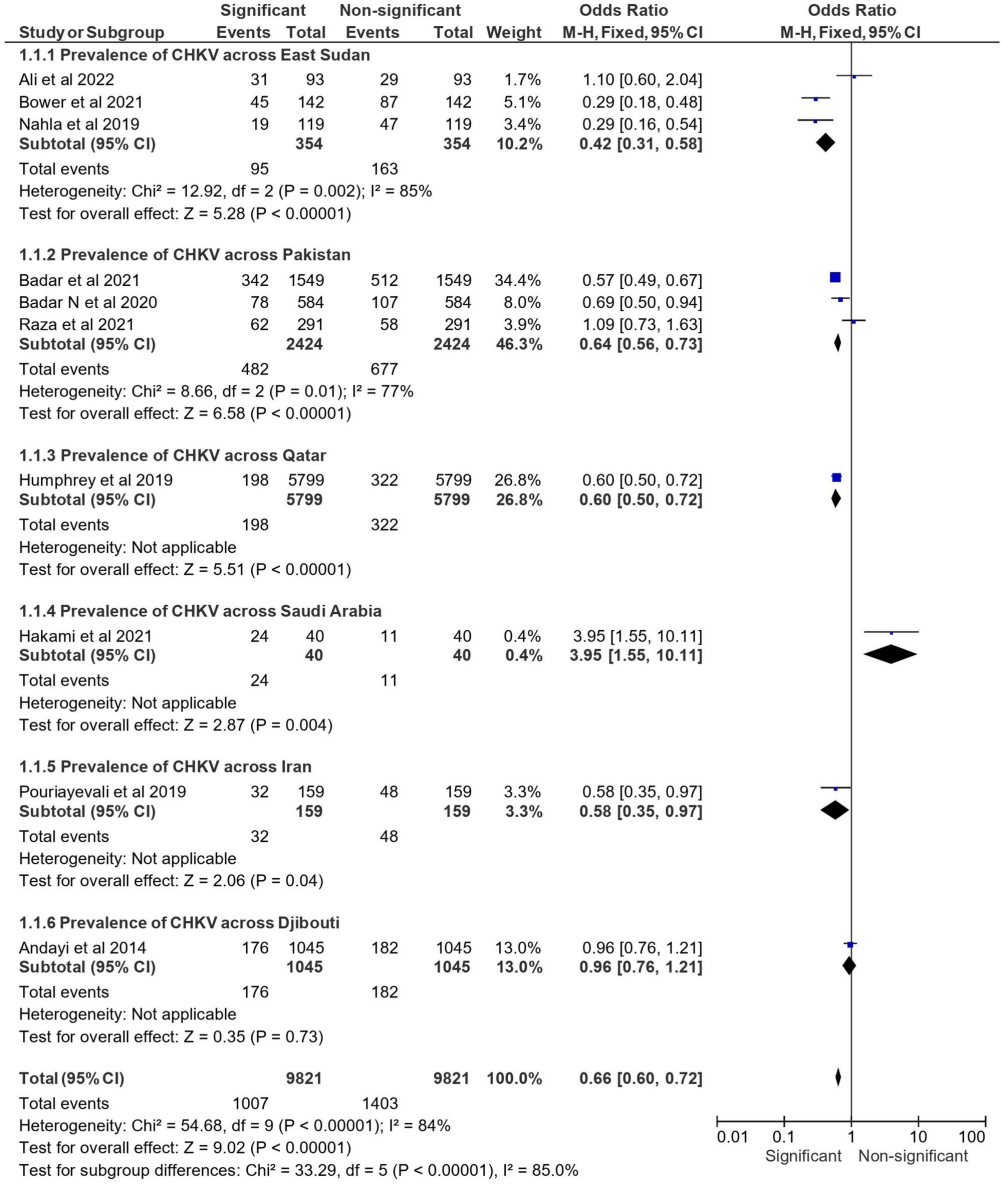
Prevalence of CHIKV across different countries in the EMR in terms of the assessed OR.

Figure 1 outlines the ORs for CHIKV prevalence in various countries, with each subcategory encompassing research on the prevalence of CHIKV within a country or area. The pooled analysis demonstrated an overall OR of 0.66 [95% CI (0.60, 0.72)]. Significant heterogeneity was indicated by an *I*^2^ of 84% and a χ^2^ of 54.68 (*df* = 9, *P* < 0.00001). The overall impact test revealed a strong association between the EMR and CHIKV prevalence (*Z*-value of 9.02, *P* < 0.00001). The test for subgroup differences also showed significant heterogeneity among the different areas, with an *I*^2^ of 85.0% and a χ^2^ of 33.29 (*df* = 5, *P* < 0.00001).

- East Sudan: 10.2% prevalence, OR 0.42 [95% CI (0.31, 0.58)]
- Pakistan: 46.3% prevalence, OR 0.64 [95% CI (0.56, 0.73)]
- Qatar: 26.8% prevalence, OR 0.60 [95% CI (0.50, 0.72)]
- Saudi Arabia: 0.4% prevalence, OR 3.95 [95% CI (1.55, 10.11)]
- Iran: 3.3% prevalence, OR 0.58 [95% CI (0.35, 0.97)]
- Djibouti: 13.0% prevalence, OR 0.96 [95% CI (0.76, 1.21)]

### CHIKV versus other ABV analysis

**Figure 2.**
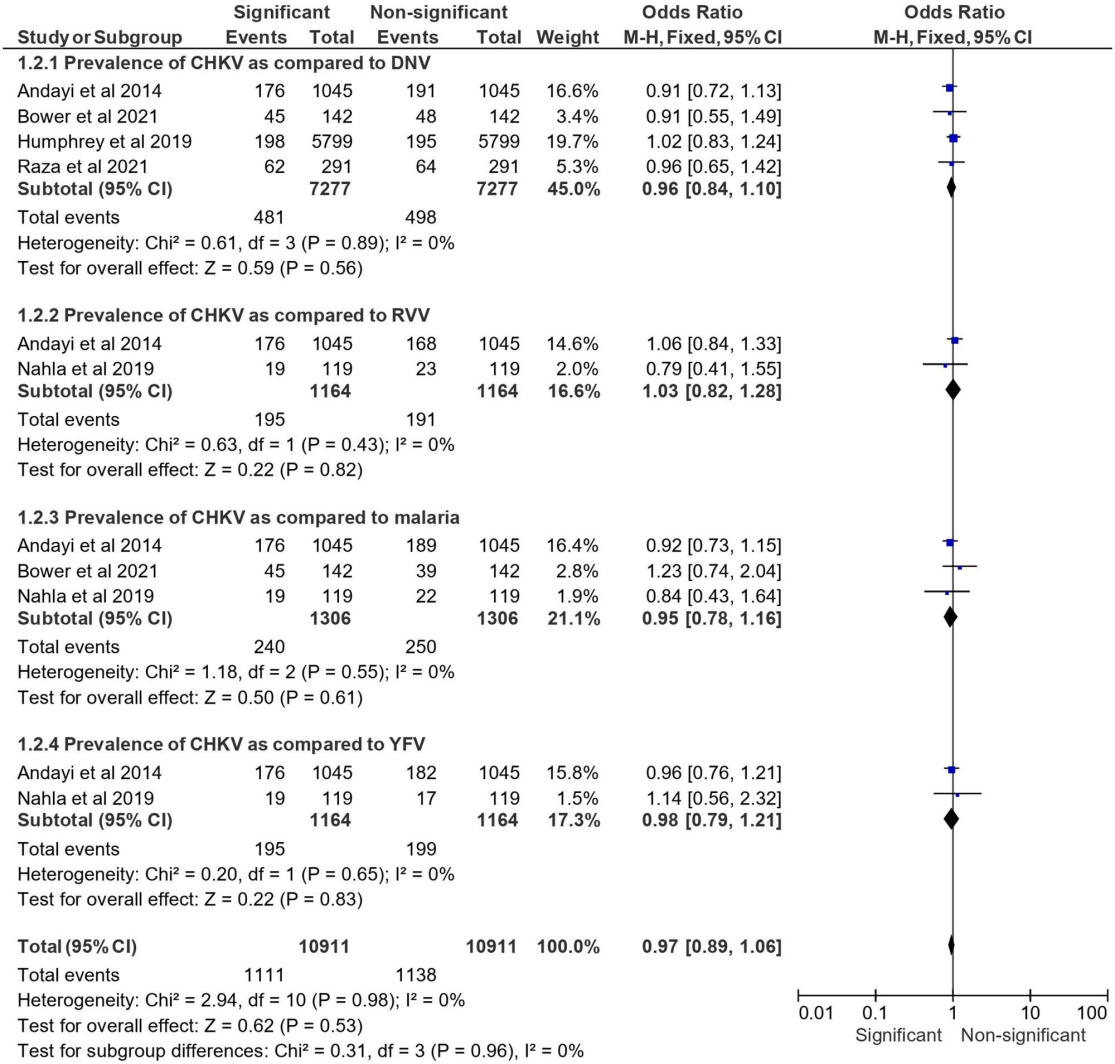
Prevalence of CHIKV serotypes in comparison to other ABVs in terms of the assessed OR.

Figure 2 presents a forest plot comparing the prevalence of CHIKV to other ABVs such as DENV, RVFV, Malaria, and YFV. The comparative analysis shows no statistically significant differences, indicating similar prevalence rates between CHKV and other ABVs. The ORs were close to 1.00 in all comparisons, and the CIs were narrow, overlapping 1.00, which indicates a lack of significant association. CHIKV versus DENV: 45.0% prevalence, OR 0.96 [95% CI (0.84, 1.10)].

- CHIKV versus RVFV: 16.6% prevalence, OR 1.03 [95% CI (0.82, 1.28)]
- CHIKV versus malaria: 21.1% prevalence, OR 0.95 [95% CI (0.78, 1.16)]
- CHIKV versus YFV: 17.3% prevalence, OR 0.98 [95% CI (0.79, 1.21)]

## Discussion

Our analysis of demographic characteristics in the selected studies provided insights helpful for determining the generalizability of findings across geographical locations and age groups. Our forest plot analysis was essential in graphically representing ABV transmission prevalence and dynamics in the EMR by comparing different ABVs and examining ORs and CIs across countries. This analysis revealed variations in CHIKV prevalence among EMR countries. The study design, systematic review, and meta-analysis approach allowed for the inclusion of research dating back to 2005, making the findings unique and significant. The study’s importance lies in its potential to influence public health policies and initiatives within the EMR by providing an understanding of ABV and CHIKV transmission dynamics and burden. This information can be used by researchers, policymakers, and healthcare professionals to develop targeted treatments, preventive measures, and surveillance systems. The study’s findings contribute to the global understanding of ABVs, as the EMR encompasses diverse demographic groups and geographical areas.

### Challenges in CHIKV prevention and response

There is little question that the CHIKV presents serious obstacles for the EMR, especially given the region’s often outdated healthcare systems [19–30]. The limited capacity for surveillance and diagnosis presents another challenge [31,32]. Healthcare systems usually lack the diagnostic tools and infrastructure needed to quickly identify and validate CHIKV cases [33]. These deficiencies complicate efforts to determine the actual cost of the illness and to quickly implement preventative measures [34]. Prophylactic measures include elimination of breeding sites for mosquitoes, personal protective measures, such as insecticide and repellent-based measures, and strategies regarding vector control. Regarding vaccines, multiple CHIKV vaccines are under various stages of development while a couple have been licensed for use in select countries and others are still being tested in the clinical setting. Similarly, vaccines for other ABVs, such as DENV and YFV, also exist and are well established and provide prevention of disease transmission [33–38].

### Comparative analysis with other diseases

Behzadi et al. [15] shift the focus from CHIKV to other vector-borne diseases, specifically those transmitted by arachnids such as ticks, in EMR countries. They report on the frequency of Q fever, Lyme disease, and tularemia, with Q fever being the most prevalent. While both Behzadi et al. [15] and our study identify significant public health concerns related to vector-borne diseases in the EMR, the vectors and diseases in question are different. The differences in vectors and diseases highlight the multifaceted nature of public health challenges in the region, encompassing a range of pathogens and transmission modes. Both studies, however, point to the need for enhanced preventive strategies and underscore the diverse epidemiological landscape shaped by the spread of various infectious diseases in the region.

Additionally, our study and the findings by Skalinski et al. [34] investigated the seroprevalence of CHIKV but within different contexts. While their review focuses on a global scale across World Health Organization regions, our study specifically examines the seroprevalence in the EMR. They reported a pooled seroprevalence estimate of 24% for all ages, with notable disparities between adults (21%) and children (7%). Our findings indicate a variable seroprevalence in the EMR, with rates as high as 46.3% in Pakistan.

### Limitations and future research

While our review synthesizes extensive data, the inherent heterogeneity in study methodologies, population demographics, and geographic settings poses challenges to the uniformity and extrapolability of our findings. The temporal and spatial specificities of the included studies may limit the applicability of the insights, suggesting a need for research to validate these findings across broader contexts. The transferability of the results, as disease prevalence and transmission dynamics may be heavily influenced by factors such as local geography, socioeconomic status, education levels, and cultural practices.

## Conclusion

We were unable to find any statistically significant difference in the prevalence of CHIKV compared to other ABVs within the EMR, meaning that the occurrence of CHIKV is not different from them. However, there are considerations of heterogeneity indicated between studies with a great amount of variation, indicating that there may be some differences in the prevalence between the member states of the EMR. Thus, the heterogeneity deserves further investigation.

## Supplementary Material

ckae165_Supplementary_Data

## Data Availability

The data generated from this study is presented as tables/figures and supplementary files. Key points The included studies spanned several countries in the EMR, with sample sizes ranging from 40 to 5799 participants. The average age of participants ranged from 27 to 36.6 years, and gender distribution varied across the studies. The reported prevalence of CHIKV varied significantly across the region, from 0.7% to 84.5%, influenced by factors such as population characteristics, local circumstances, and assessment techniques like ELISA and RT-qPCR. A pooled analysis demonstrated an overall OR of 0.66 [95% CI (0.60, 0.72)], indicating a strong association between the EMR and CHIKV prevalence. Significant heterogeneity was observed across the region, with an *I*^2^ of 84%. Regional differences in CHIKV prevalence were evident, with notable prevalence rates in Pakistan (46.3%), Qatar (26.8%), and East Sudan (10.2%), while Saudi Arabia had the lowest reported prevalence of 0.4%. Comparative analysis with other ABVs such as DENV, RVFV, malaria, and YFV showed no statistically significant differences, suggesting similar prevalence rates for CHIKV and other ABVs.
