# Supplementary material for: Evaluating the burden and transmission dynamics of chikungunya virus infections in the Eastern Mediterranean Region: a systematic review and meta-analysis
Source: Eur J Public Health. 2025 Jan 13;35(Suppl 1):i27–34. doi: 10.1093/eurpub/ckae165 (PMC11725947; doi:10.1093/eurpub/ckae165)

**List of Supplementary Tables and Figures**

***Manuscript***: Evaluating the Burden and Transmission Dynamics of Chikungunya Virus Infections in the Eastern Mediterranean region: A Systematic Review and Meta-Analysis

**Supplementary Table 1:** Search Strings Utilised across Databases

| Database | Search string | MeSH/Boolean Operator | Cutoff Date for Publication |
| --- | --- | --- | --- |
| PubMed | ("Chikungunya"[MeSH Terms] OR "Chikungunya Virus"[MeSH Terms] OR "Chikungunya Fever"[MeSH Terms]) AND ("Eastern Mediterranean Region"[MeSH Terms] OR "Middle East"[MeSH Terms] OR "North Africa"[MeSH Terms]) AND ("Disease Transmission"[MeSH Terms] OR "Epidemiology"[Subheading] OR "Incidence"[MeSH Terms]) AND ("2000/01/01"[Date - Publication]: "2023/06/30"[Date - Publication]) | MeSH Terms, AND, OR, Subheadings | June 2023 |
| Embase | ('Chikungunya virus infection'/exp OR 'Chikungunya fever'/exp) AND ('Middle East'/exp OR 'North Africa'/exp) AND ('Disease transmission'/exp OR 'Virus epidemiology'/exp) AND [2000-2023]/py | Emtree Terms, AND, OR |  |
| Web of Science | TS=((Chikungunya OR "Chikungunya Virus" OR "Chikungunya Fever") AND ("Eastern Mediterranean" OR "Middle East" OR "North Africa") AND (Transmission OR Epidemiology OR Incidence)) AND PY=(2000-2023) | Topic Search, AND, OR |  |
| Scopus | (TITLE-ABS-KEY (Chikungunya) OR TITLE-ABS-KEY ("Chikungunya Virus") OR TITLE-ABS-KEY ("Chikungunya Fever")) AND (TITLE-ABS-KEY ("Eastern Mediterranean") OR TITLE-ABS-KEY ("Middle East") OR TITLE-ABS-KEY ("North Africa")) AND (TITLE-ABS-KEY (Transmission) OR TITLE-ABS-KEY (Epidemiology) OR TITLE-ABS-KEY (Incidence)) AND (PUBYEAR AFT 1999 AND PUBYEAR BEF 2024) | Boolean Operators, AND, OR |  |
| Cochrane Library | #1 MeSH descriptor: [Chikungunya] explode all trees AND #2 MeSH descriptor: [Disease Transmission] explode all trees AND #3 (Middle East OR Eastern Mediterranean OR North Africa) AND Year of publication from 2000 to 2023 | MeSH Descriptors, AND, OR |  |
| CINAHL | (MH "Chikungunya Virus+") AND (MH "Disease Transmission+") AND (JN "Eastern Mediterranean" OR JN "Middle East" OR JN "North Africa") AND (DT "20000101-20230630") | CINAHL Headings, AND, OR |  |
| PsycINFO | AB (Chikungunya OR "Chikungunya Virus" OR "Chikungunya Fever") AND (AB "Eastern Mediterranean" OR AB "Middle East" OR AB "North Africa") AND (AB Transmission OR AB Epidemiology OR AB Incidence) AND (PY=2000-2023) | Boolean Operators, AND, OR |  |
| ScienceDirect | KEY(Chikungunya AND ("Eastern Mediterranean" OR "Middle East" OR "North Africa")) AND (KEY(Transmission) OR KEY(Epidemiology) OR KEY(Incidence)) AND PUBYEAR AFT 1999 AND PUBYEAR BEF 2024 | Boolean Operators, AND, OR |  |

**Supplementary Table 2:** Inclusion and Exclusion Criteria for the Review

| Criteria type | Inclusion standards | Exclusion criteria | PICOS framework |
| --- | --- | --- | --- |
| Study Types | Epidemiological and prospective/retrospective studies | Non-epidemiological study designs not providing evidence of CHKV infections in humans | Epidemiological and prospective/retrospective studies focusing on CHKV frequency, distribution, and risk factors |
| Documentation | Documentation of human cases of CHKV or genetic analysis of the virus | Studies without human case documentation of CHKV infections or genetic analysis of CHKV | Population: Individuals with CHKV infections in the EMR region |
| Evaluations | Evaluations of CHKV using molecular methods, genetic sequencing, serological testing, or laboratory diagnostic procedures | - | Intervention/Exposure: CHKV infection or its genetic analysis |
| Statistics | Statistics related to the evaluated human population, such as comorbidities, co-circulation with other arboviral illnesses, prevalence rates, or transmission dynamics | Studies providing only geographic information without specific statistics on the assessed human population | Outcomes: Prevalence of CHKV infection, transmission dynamics, associated comorbidities, co-circulation with other arboviruses |
| Exclusions | - | Prospective/retrospective studies, and other non-epidemiological study designs were eliminated from consideration. | Comparison: Different regions and their respective incidence of CHKV in the EMR and comparison of the other arbovirus incidence with CHKV |

**Supplementary Table 3:** GRADE Assessment of Study Quality and Risk of Bias

| Study design | Number of studies | Observed common finding | Risk of Bias | Inconsistency | Indirectness | Imprecision | Others | Certainty |
| --- | --- | --- | --- | --- | --- | --- | --- | --- |
| Cross-sectional | 9 | Various outcomes related to CHKV infection, including pregnancy complications and co-circulation with other arboviruses | Low | Low to moderate | Low | Low to moderate | None reported | Moderate |
| Prospective cohort | 1 | No viral RNA found in most specimens; quick recovery from CHKV | Low | Low | Low | Low | None reported | Moderate |

**Supplementary Figure 1:** Article selection framework in terms of the PRISMA guidelines as utilised in this investigation.


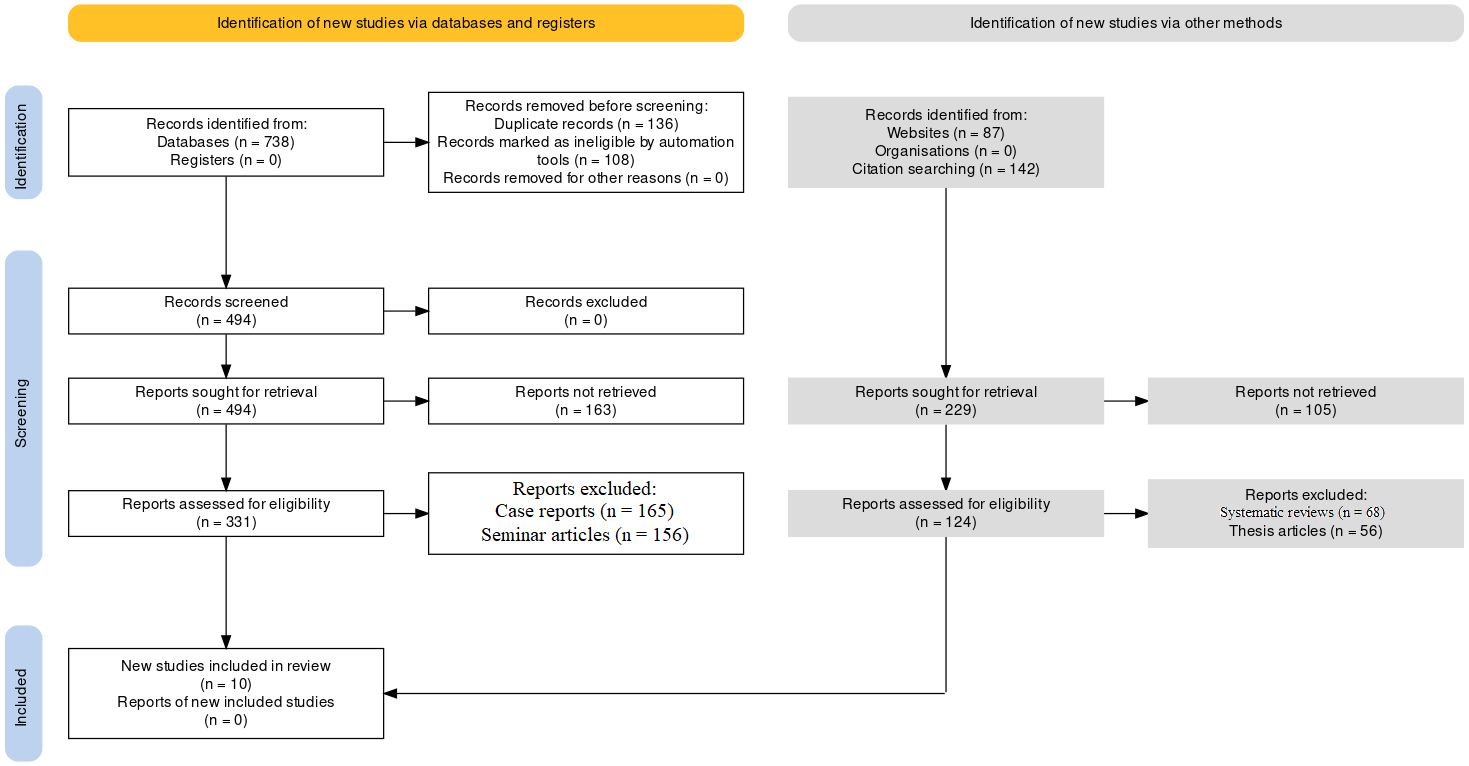


- **Supplementary Figure 2:** Evaluation of bias in the selected cross-sectional articles selected for the review.


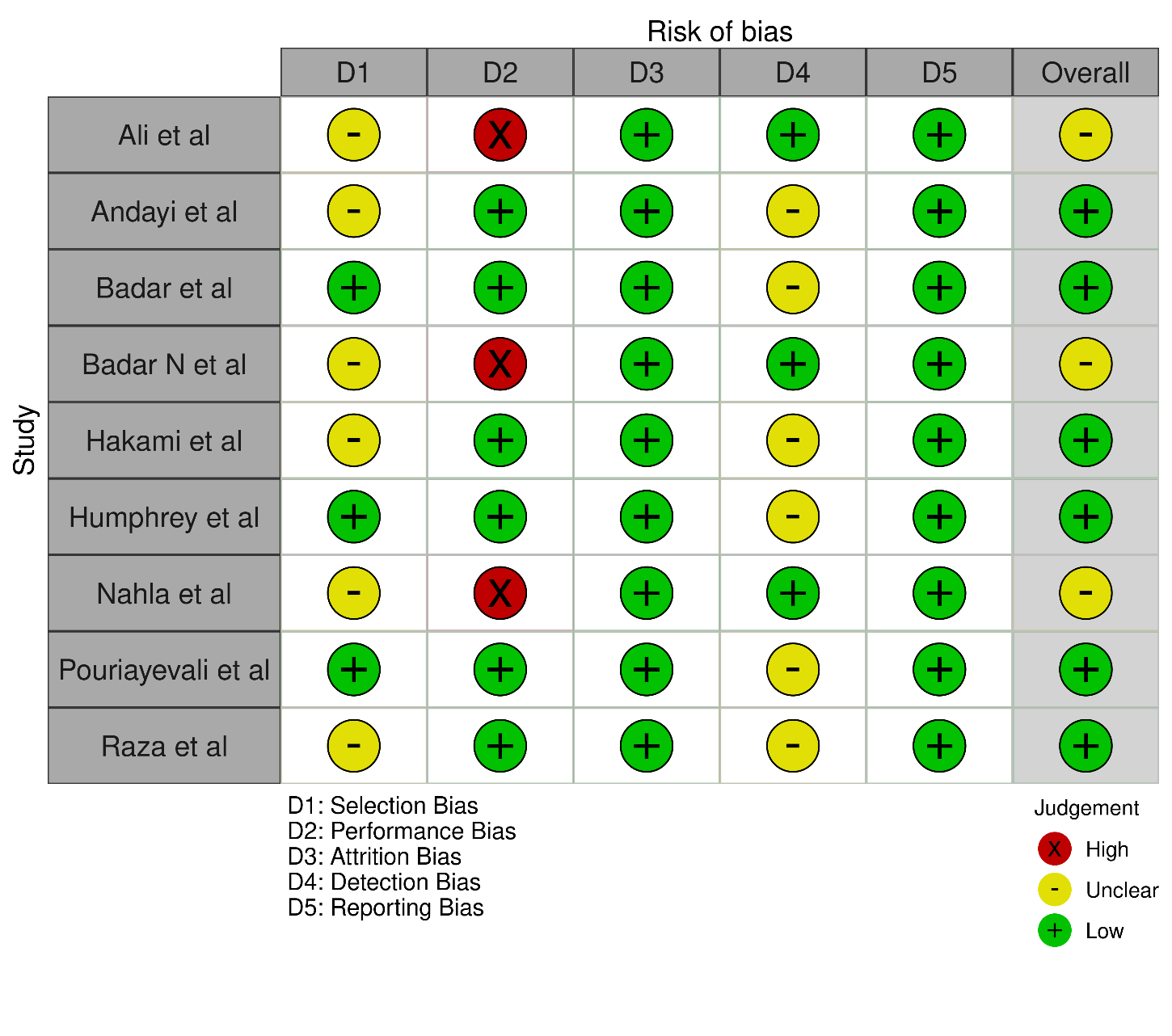


- **Supplementary Figure 3:** Evaluation of bias in the cohort-based papers selected for the review.


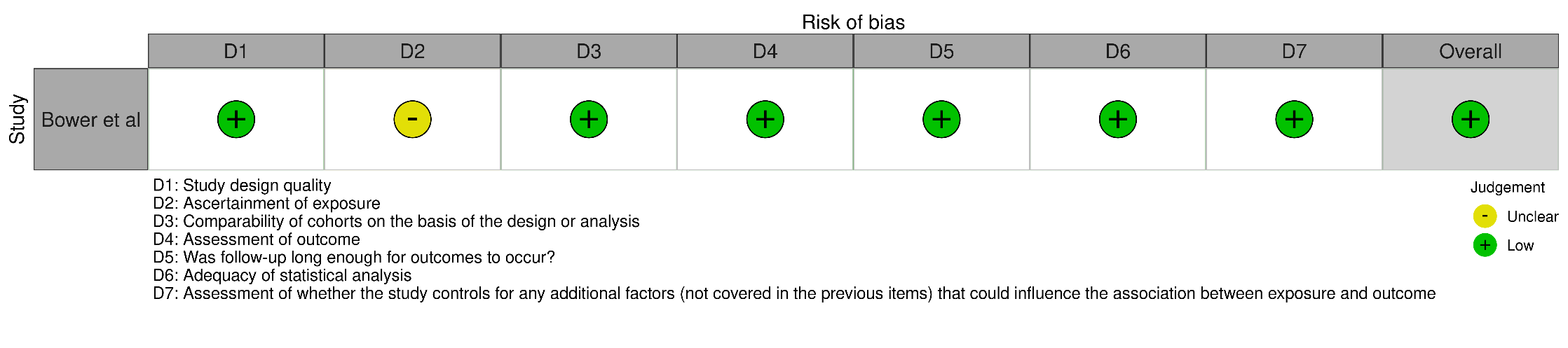

Supplement: ckae165_Supplementary_Data [file ckae165_supplementary_data.docx]
